# Hemolytic uremic syndrome following the infusion of oxaliplatin: case report

**DOI:** 10.1186/1472-6904-6-5

**Published:** 2006-09-12

**Authors:** Issa Dahabreh, George Tsoutsos, Dimitrios Tseligas, Dimitrios Janinis

**Affiliations:** 1 Department of Medical Oncology, Athens Medical Center, Marousi, Greece; 2 Department of Surgery, Athens Medical Center, Marousi, Greece

## Abstract

**Background:**

Oxaliplatin is a platinum derivative, which is used in the treatment of colorectal cancer. A small number of oxaliplatin-related hemolytic and/or thrombocytopenic reactions have been reported. We present a case of hemolytic-uremic syndrome that developed during the 4^th ^cycle of combination chemotherapy with oxaliplatin, 5-fluorouracil and leucovorin.

**Case presentation:**

A 52-year-old-male was administered chemotherapy with oxaliplatin, 5-fluorouracil and leucovorin for a Duke's stage C colorectal carcinoma. Three cycles of chemotherapy had been administered without complications when, at the beginning of the fourth cycle, the patient developed clinical and laboratory abnormalities consistent with the development of the hemolytic-uremic syndrome. Treatment was discontinued; the patient was managed with monitored IV hydration and loop diuretics, high dose corticosteroids and fresh frozen plasma infusions and recovered completely.

**Conclusion:**

The hemolytic-uremic syndrome may be a rare complication of oxaliplatin-based chemotherapy. Clinicians need to maintain a high index of suspicion to diagnose and treat this life-threatening adverse event.

## Background

Oxaliplatin (L-OHP) is a third generation platinum derivative with proven effectiveness in the treatment of colorectal cancer. Combined with 5-fluorouracil (5-FU) and leucovorin as a 48 hour infusion every two weeks (FOLFOX regimen), L-OHP is drastic in the adjuvant, first and second line setting [1]. Common adverse effects of this regimen include reversible sensory neuropathy, myelosuppression and nausea [2]. Here, we describe a patient who developed acute hemolysis and thrombocytopenia after the 4^th ^cycle of chemotherapy with FOLFOX.

## Case presentation

A 52-year-old-male, with a prior history of non-insulin-dependent diabetes mellitus, underwent sigmoidectomy for a moderately differentiated adenocarcinoma of the sigmoid colon. Pathologic staging revealed a Duke's stage C tumor or C_1 _according to the Astler-Coller classification. A decision was made to administer adjuvant chemotherapy using the FOLFOX-4 regimen (5-FU, 400 mg/m^2 ^bolus infusion on days 1, 2 followed by 5-FU, 600 mg/m^2 ^as a continuous infusion for 22 hours on days 1, 2, leucovorin 200 mg/m^2 ^as a 2 hour infusion prior to 5-FU on days 1, 2 and L-OHP 85 mg/m^2 ^on day 1, every 2 weeks). He was programmed to receive a total of 6 monthly cycles. Treatment was well tolerated until the 4^th ^cycle, at the beginning of which the patient's hematologic profile and renal function were normal (Hb: 13,8 g/dl, HCT: 43%, WBC: 4,9 × 10^9^cells/L, platelets: 162 × 10^3 ^cells/μg, creatinine: 1,1 mg/dl, BUN: 35 mg/dl).

On the first day of therapy, and after L-OHP infusion was completed, the patient noticed urine discoloration and immediate urine analysis demonstrated hematuria with hemoglobinuria. During the next day, the patient's complete blood count exhibited findings consistent with acute hemolysis and thrombocytopenia (Hb: 12 g/dl, Hct: 36%, WBC: 4,78 × 10^9 ^cells/L and platelets: 50 × 10^3 ^cells/μl). A hemolytic reaction was indicated by elevated LDH and indirect bilirubin indices (1383 U/l and 3,34 mg%, respectively). The patient also exhibited signs of acute anuric renal failure (urea: 77 mg%, serum creatinine: 3,1 mg% and uric acid: 9,5 mg%). Peripheral smear analysis demonstrated fragmented RBCs.

Chemotherapy was immediately discontinued and the patient was managed conservatively with monitored IV hydration and loop diuretics, high dose corticosteroids and fresh frozen plasma (FFP) infusions on a daily basis.

The development of hemolysis was further substantiated with next day's laboratory results. LDH remained elevated at 819 U/l. Coagulation parameters were normal, partial thromboplastin time, fibrinogen and INR were 31,1 sec, 412 mg/dl and 1.05, respectively. D-dimmers were 9,1 μg/ml. The patient was not G6-PD deficient (G6-PD: 11.0 U/g Hb). Direct and indirect agglutination reactions remained negative providing no indication of immune mediated hemolysis. Hemoglobin and hematocrit values reached a minimum of 11.8 g/dl and 34.8% respectively, four days after the infusion of L-OHP. At that point, LDH was 499 U/l and BUN and creatinine were 149 mg/dl and 5,6 mg/dl, respectively.

The patient gradually recovered and the results of successive hematological and biochemical tests confirmed the improvement of his condition. Fifteen days after the onset of hemolysis and thrombocytopenia laboratory values were returning to normal: Hb: 12,1 g/dl, Hct: 38,2%, platelets: 151 × 10^3 ^cells/μl, LDH: 255 U/l, creatinine: 1,5 mg/dl, BUN: 78 mg/dl. A month later, all hematologic values and the urine analysis had returned to normal. The patient refused the continuation of chemotherapy. On follow-up, one year after the hemolytic episode, the patient remained disease free and asymptomatic, with no evidence of recurrence of hemolysis or thrombocytopenia. BUN and creatinine were 42 mg/dl and 1,2 mg/dl, respectively, indicating the absence of chronic renal sequelae.

## Discussion

The combination of L-OHP with 5-fluorouracil/leucovorin (FOLFOX) has proven efficacy in the treatment of advanced colorectal cancer [1]. Common adverse effects of this drug combination are neutropenia, diarrhea, and most importantly, reversible sensory neuropathy [2]. Literature reports of L-OHP-related acute hemolysis and/or thrombocytopenia are scarce [3-11]. The pathogenetic mechanisms underlying this condition are controversial. The present case clearly demonstrated signs of acute intravascular hemolysis associated with thrombocytopenia and renal impairment.

Three mechanisms have been considered for the pathogenesis of hemolysis with thrombocytopenia associated with L-OHP-based chemotherapy: (a) antibody mediated destruction of platelets and erythrocytes (b) microangiopathic hemolytic anemia due to drug-induced thrombotic thrombocytopenic purpura (TTP), and (c) microangiopathic hemolytic anemia due to disseminated intravascular coagulation (DIC) [9,10]. In the present case, DIC was readily excluded, since D-dimmers were only slightly elevated and aPPT and INR values were normal. Review of the literature on all reported cases of hemolysis and/or thrombocytopenia related to L-OHP, was negative for the presence of DIC.

Antibody mediated hemolysis and/or thrombocytopenia is considered the most common etiology for a clinical presentation similar to that exhibited by our patient. An immune-complex type hemolytic anemia has been postulated as a possible pathogenetic mechanism [5]. Hemolysis provoked by the adsorption of L-OHP on the surface of red blood cells (RBCs) has also been considered a probable cause or contributing factor [3,8]. Such a phenomenon is invariably associated with a positive direct agglutination test (direct Coomb's reaction), which was not present in our case. Moreover, the presence of antibodies against both the RBCs and the platelets (commonly termed the "Evans' syndrome") is a rare cause of drug induced hemolysis and thrombocytopenia, and there have been few reports of L-OHP induced Evan's syndrome in the literature [6,9].

The hemolytic uremic syndrome (HUS) and TTP are the most common manifestations of a group of disease states termed "thrombotic microangiopathies". The distinction between these two conditions is difficult on clinical grounds [12]. A classic pentad of signs has been associated with TTP: microangiopathic hemolytic anemia, thrombocytopenia, neurological abnormalities, renal failure and fever. In practice, the combination of thrombocytopenia and hemolytic anemia, in the absence of any apparent alternative cause is enough to suggest the presence of TTP or HUS. If renal failure dominates the clinical picture, then the disorder is considered the HUS [13-15].

Our patient exhibited laboratory values consistent with the HUS (hemolytic anemia, elevated LDH, normal coagulation parameters, negative findings on the DAT, and acute renal failure). A number of drugs have been implicated as causal agents of thrombotic microangiopathy, either as an acute, immune-mediated toxicity (quinine, ticlodipine, clopidogrel) or as a cumulative, dose-dependent toxicity (mitomycin-C, gemcitabine) [12-18]. The sudden onset of hemolysis and thrombocytopenia in our patient seems to favor an immune-mediated reaction. Any of the administered drugs could have been responsible for this reaction. Development of the HUS has been reported as a severe toxicity of 5-FU/leucovorin chemotherapy, almost always in combination with mitomycin-C or other agents known to cause HUS/TTP [18-21]. It has been suggested that 5-FU/leucovorin may increase the incidence of mitomycin-C induced HUS. We cannot exclude the occurrence of such a synergistic effect in our patient. Since no further 5-FU-based chemotherapy was administered, any potential role of 5-FU/leucovorin in the development of HUS cannot be definitively ruled out. To our knowledge, this is the first case of L-OHP-related HUS reported in the literature.

The treatment of drug related HUS remains controversial [15,16,18]. Mainstays of treatment include the discontinuation of the offending agent, supportive care and FFP infusion [13-16]. Some investigators favor plasma exchange to FFP infusions, although the superiority of either method in the management of this patient population has not been proven [15]. In the present case, response was favorable to conservative management.

## Conclusion

Acute hemolysis, with or without thrombocytopenia, is a rare but potentially life-threatening complication of L-OHP-based chemotherapy. We described the case of a patient who developed the HUS after the 4^th ^cycle of FOLFOX chemotherapy and was successfully managed. In view of the relatively recent approval of L-OHP from the FDA (August, 2002), and the expected broad application of this agent in a variety of solid tumors [22], the incidence of acute hematological events related to its use will likely increase. Thus, increased vigilance on behalf of the oncologic community is required.

## Competing interests

The author(s) declare that they have no competing interests.

## Authors' contributions

ID participated in the case report design, interpreted patient data and drafted the manuscript. GT and DT participated in the case report's design. DJ conceived of the study, participated in its coordination and proofed the manuscript. All authors read and approved the final manuscript.

## Pre-publication history

The pre-publication history for this paper can be accessed here:


